# Increasing systematicity leads to better selection decisions: Evidence from a computer paradigm for evaluating selection tools

**DOI:** 10.1371/journal.pone.0178276

**Published:** 2017-05-22

**Authors:** Martin Bäckström, Fredrik Björklund

**Affiliations:** Department of Psychology, Lund University, Lund, Sweden; Mälardalen University, SWEDEN

## Abstract

A computerized paradigm was created to allow for testing in the laboratory whether increasing systematicity helps the recruiter make better selection decisions. Participants were introduced to the job and the applicants on the computer screen and asked to select who they thought should be considered for the job and who should not. Level of systematicity, i.e. the extent to which the recruitment is methodical and uses prepared tools, was manipulated between subjects. Depending on experimental condition participants were helped by means of a tool for extracting judgment criteria (job analysis) and a tool for making judgments related to selected criteria (including calculation of a final score). The general prediction that increased systematicity leads to the selection of more qualified candidates was supported by the results, particularly when the motivation to put time and effort into the task was higher. The results support the claim from Industrial/Organizational psychologists that systematicity is a desirable characteristic in selection processes. The fact that increasing systematicity led to better selection decisions in a controlled laboratory experiment, along with process-related measures, suggests that this kind of paradigm could be useful when evaluating new tools for improving selection decisions, before they are tested in large (and costly) field studies of actual personnel selection.

## Introduction

How should we design the selection process so that it provides maximal support to the recruiter in choosing the best candidates? Our short answer is that the process should be characterized by systematicity, i.e. working in a methodical fashion using prepared tools. For each tool that the recruiter uses in the intended way the selection quality should increase. The present research will show that tools help the recruiter select the best applicants from a pool of candidates that at first glance may seem equally competent. Personnel selection usually involves several applicants that differ on a number of more or less job-relevant qualities and it is no trivial task to filter out the job-relevant information and weight it to form a well-founded decision. Therefore support tools, properly used, may lead to better selection decisions.

Systematic personnel selection has been advocated by Industrial/Organizational psychologists for years [1]. However, the related research has not probed the incremental effects of decision tools on outcome measures, such as the quality of the chosen candidates, using experimental methodology. Perhaps one reason for this is the restrictions that the otherwise laudable field studies may put on the possibilities of evaluating the effects of raising the level of systematicity on outcomes, and another the cost in time and effort that this kind of studies involve. It is our hope that the present research, introducing a less costly, laboratory-based, method for evaluating selection-related decision making tools, might complement the field research. Our methodological niche is in between applied research and cognitively oriented research, and allows for studying process related variables in a context that is relevant for selection researchers.

### An overview of psychological research on selection

In psychology, personnel selection has been a research topic for at least a century [2]. Much of the research has concerned measurable individual differences in job related constructs, and which selection methods provide the most productive candidates. A great many things have been established, not least regarding the criterion validity of different methods, i.e. the extent to which they correlate with actual job performance. Since the fifties, researches have emphasized the importance of psychological testing and calculation tools, and some have expressed dissatisfaction with the fact that professional recruiters still rely highly on the (often unstructured) employment interview and holistic judgments of the gathered data [1]. Overall, field studies have provided evidence suggesting that a systematic approach to selection, i.e. a structured process that emphasizes validated instruments and methods rather than experience and gut-feeling, is to be preferred [3–5].

There now appears to be a relative consensus in the selection research area concerning how the personnel selection process should ideally be designed. In principle, it is recommended that everything from needs analysis, job analysis, choice of selection instruments, usage of the instruments and evaluation of the results, information gathering, decision making, to evaluation of the process itself, should be characterized by a systematic, analytical approach. In other words, a good selection process should involve careful job analysis, relate the job content to specific criteria for judgment, collect information with elaborated methods (structured interviews, weighed application papers and different knowledge tests) and base decisions on a transparent algorithm for combining the gathered data. These ideas regarding how an ideal selection process is to be conducted is conveyed in the *Principles for the validation and use of personnel selection procedures* from division 14 (Society for Industrial and Organizational Psychology) of the American Psychological Association, and in the ISO-standard 10667 *Assessment service delivery–Procedures and methods to assess people in work and organizational settings*. Both documents are widely spread, but do not appear to have had the impact on actual selection processes that one might have hoped. Actual selection processes are characterized by clear divergences from systematicity (the “academic-practitioner gap”, [1, 6–8]).

### Aims and scope of the present study

The present study does not concern why personnel selection practices often diverge from scientists’ recommendations. Rather, it concerns the degree to which different aspects of systematicity actually promote identification of the best candidates for a job. The primary focus is on how decision making can be supported. We expect increased systematicity to be linked to better selection. As opposed to previous research on systematicity and selection, we attempt to experimentally manipulate the level of systematicity and measure its effects on decision quality. This is done by adding specific tools designed to help the recruiters work in a more systematic fashion. One tool is for extracting judgment criteria (job analysis) and one is for making judgments related to selected criteria (including calculation of a final score). The goal is to investigate whether the recruiter is well advised to use the tools, with regard to the outcome of the process. In other words, do the tools help the recruiter make better selection decisions? We predict that the effect of systematicity (up to a point) is approximately linear, which in this case would mean that having access to no tools produces the worst selection decisions and having access to both tools produces the best selection decisions.

Our methodological approach differs from the usual in selection research; instead of field studies we perform experimental studies in the laboratory. The experimental task is set up to resemble an actual selection task. It is more elaborate and involved than the usual studies in e.g. social psychology, where participants are often provided with CV:s and asked to read them and make hireability judgments. Instead our participants work actively with different aspects of the selection process. To the extent that increased systematicity leads to selecting more qualified candidates, the validity of systematic selection processes is supported.

In addition to our overall prediction that systematicity will increase the likelihood of selecting the most qualified candidates for the job at hand, we expect the benefits of systematicity to be larger when the tools are used in the way that they were designed to be used. Systematic selection is taxing, and those who are motivated to perform the task carefully should make better selection decisions. Furthermore, since we vary the degree of systematicity it will be possible to see whether hybrid forms of systematicity and more intuitive thinking are superior to pure systematicity, which is not predicted by us but has been suggested by others to be the case at least under some circumstances [8]. To provide some background and theoretical framework for the study, the research literature regarding the importance of job analysis and worker attribute inferences will now be reviewed.

### Recruiting systematically

Ratings of applicants can be made using either a holistic or a decomposed judgment strategy. Holistic ratings are global, univariate and provide an overall assessment. Decomposed ratings concern several different aspects (specifics that can be combined later). Decision researches generally see merits in breaking down judgments into components, e.g. [9–10] but the issue is still controversial. Once ratings have been made they need to be combined into an overall assessment for each candidate, to enable a selection decision. Data can be combined either mechanically (i.e. systematically, based on an algorithm) or holistically (i.e. intuitively). Meta-analyses of studies comparing mechanical and holistic combination have consistently shown that mechanical models are superior in predicting job performance [11]. This would suggest that mechanical data combination is associated with better selection decisions too, although notably selection decisions and job performance predictions are not the exact same thing.

For the mechanical data combination to be successful, the data needs to be based on valid information. In this regard, a job analysis is a necessary preparation tool, enabling the decisions to be based on valid information. Research on job analysis shows that differentiating the ratings such that they concern the specific tasks that the job involves rather than the job as a whole generally improves judgments [12], particularly after training, as evidenced in effects of frame-of-reference training on the quality of competence modeling ratings [13]. Similarly, job analysis training reduces the tendency of the recruiter to overvalue those of his or her own personality traits that are not related to the job per se [14]. These findings are very relevant for the present purposes and tentative evidence for the importance of job analysis in selection, therefore we have included job analysis in our experimental set up. Notably, however, the outcome measures in previous research on job analysis have concerned tasks and traits in general, rather than applicant appraisal or selection decisions.

It should also be noted that recruiters are unlikely to have an algorithm for calculating (raw) data from an interview or a CV. Instead, in this situation people are likely to vary in how systematic ratings and interpretations they make. In support of this contention it has been found that perceived level of expertise [15], degree of involvement in the model’s development [16] the effects that the decisions may have [17] as well as the mere preference to base decisions on intuitions and feelings [18] all affect recruiter’s willingness to rely on calculation-based decision aids. In the present study, the recruiters will either be provided with a rating tool that supports them in making inferences regarding the applicants’ attributes (decomposed judgments) or will not be provided with a rating tool. We expect participants who are provided with a rating tool to be better at discriminating the applicants on their actual level of qualification, such that the candidates that they select are more suited for the job than those selected by participants who are not provided with a rating tool.

### Ethics statement

The studies in this report were approved by the Regional Ethical Review Board in Lund (EPN; Lund, D.nr. 2009–3). Participants received verbal and written information about the study before signing when consenting to participate.

## General method

### The computer application

The present research will show how factors affecting selection decisions can be studied by means of a computer application. The application was developed and pre-tested in several steps, fine-tuning the job description, the variability among the applicants’ qualifications in relation to the job description, as well as the number of applicants, etc. (for requesting the application, which was developed in Windows Presentation Foundation, please contact the first author).

In all studies of the present paper, participants’ task was to make a selection of job applicants. Before the selection they processed the information about the applicants. They were randomly assigned to one of the following conditions (levels of increasing process structure):

- Level 0: Unsystematic—reading a short advertisement about the job.
- Level 1: Somewhat systematic—reading a job description of the job.
- Level 2: Systematic—reading the job description and responding to job analysis items, the mean ratings on job-related criteria being displayed on the screen.
- Level 3: Highly systematic—as systematic, but also rating each applicant on the job related criteria while reading the CVs.

The computer application provided:

1. General instructions.
2. Information about the job
  - An advertisement or a job description
  - A job analysis tool (in the systematic conditions)
3. The applicants and their CVs.
4. A module where applicants could be selected.

The computer application (http://www.pimahb.com/selectiondemo.mp4 or http://www.pimahb.com/selectiondemo.avi for a demonstration) was created to automatically present all information to the participants, and lead them through the entire experimental procedure. Having the computer controlling the procedure enabled testing of several participants in the same room, and measuring the time it took them to complete different parts of the procedure. Another advantage was that it made the selection procedure more dynamic and involving. Participants had the possibility to choose an applicant they were interested in and read about his or her qualifications. If they felt that an applicant was highly qualified, they could put that applicant aside as a candidate for the job. They could return to the applicants several times, and make changes to ratings and selections. Furthermore, the computer application helped the participants by indicating which applicants they had already read about.

**Fig 1:**
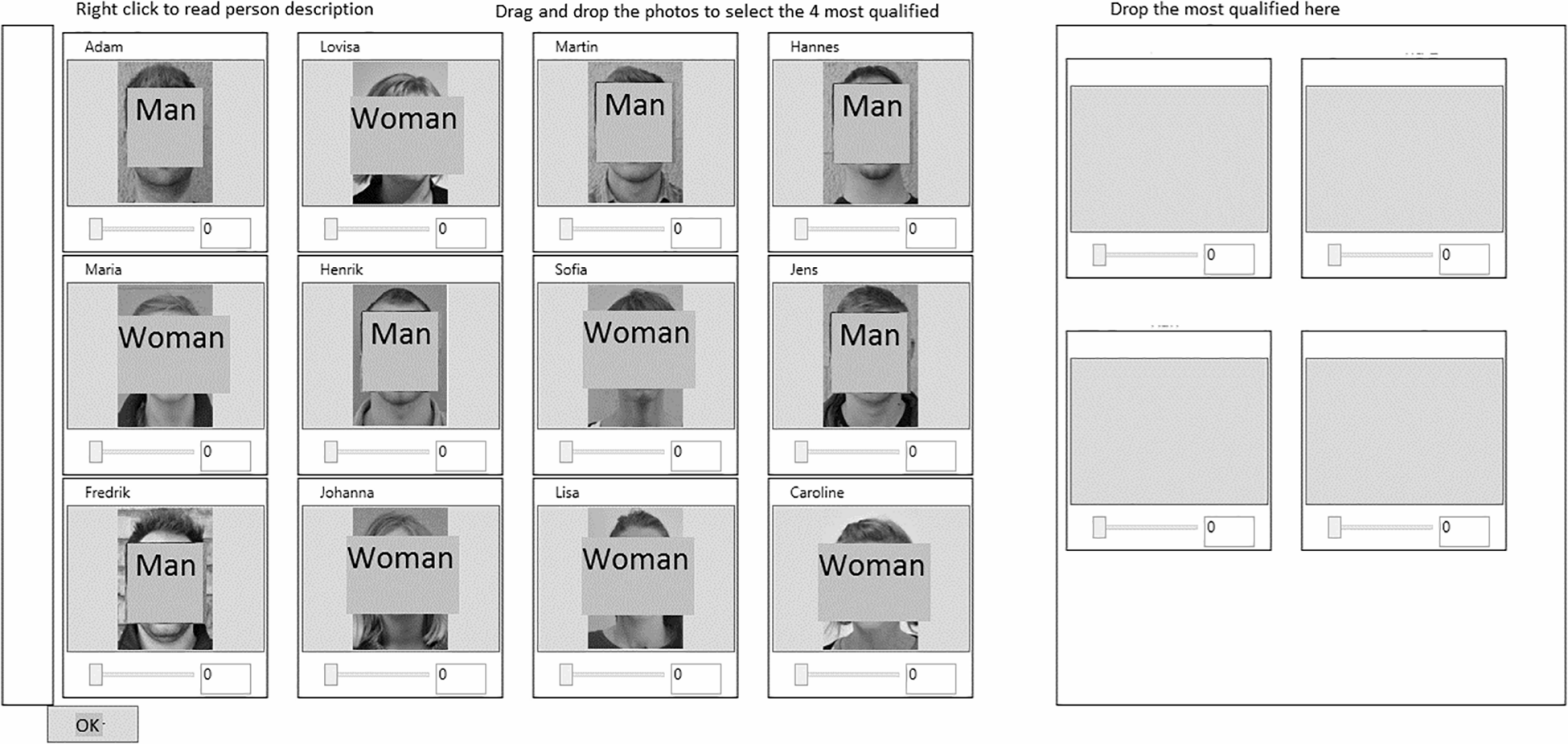
The selection module used in the application (pictures covered to anonymize).

**Fig 2:**
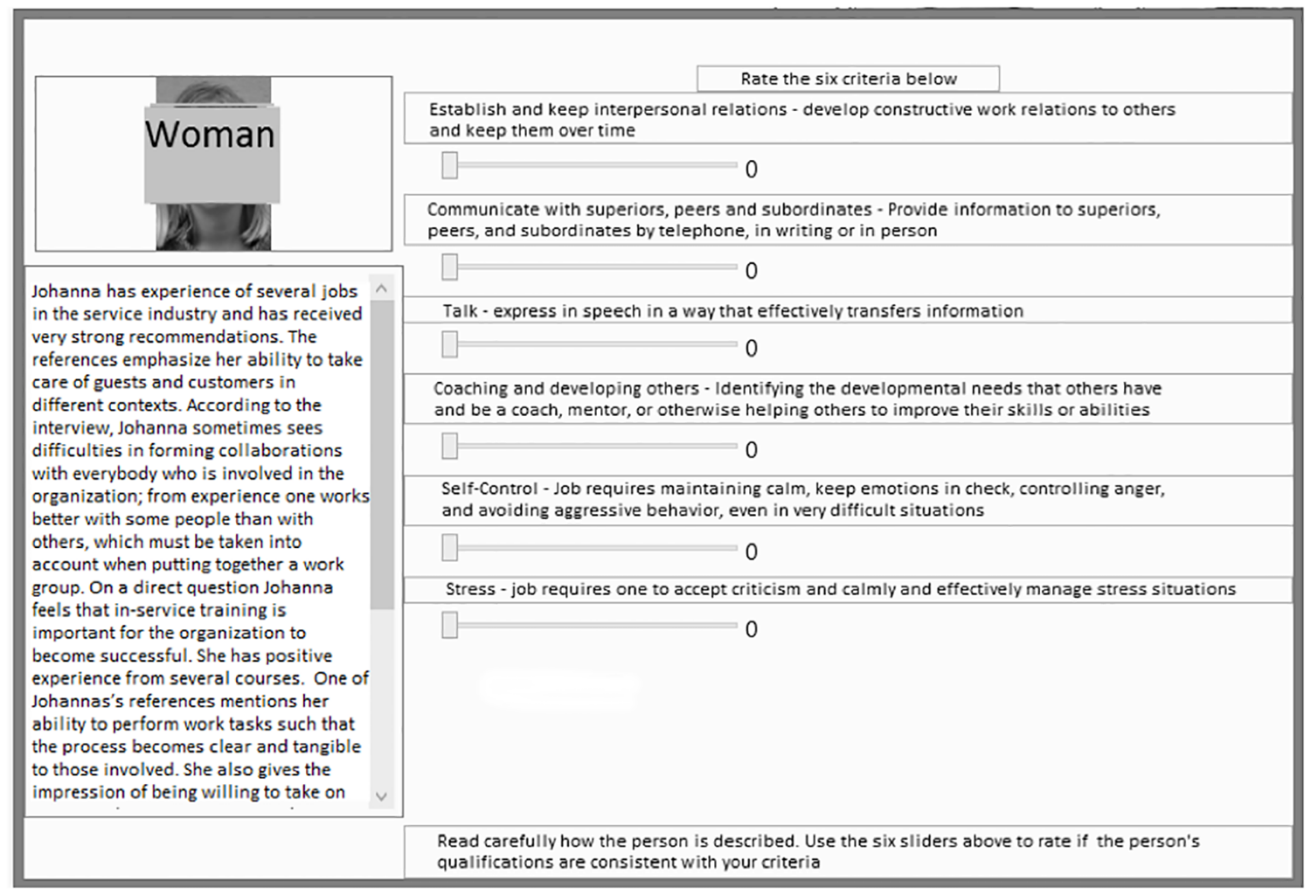
The CV reading and rating interface used in the highly systematic condition (picture covered to anonymize).

The selection was performed by dragging the applicants’ photographs to a special section in the rightmost part of the screen (Fig 1). In the highly systematic condition a rating tool was added to the module where participants read the CVs (Fig 2). The rating tool consisted of 6 sliders with a 0–10 scale, related to the criteria from the job analysis. Participants rated the applicants on the criteria and the computer application paged them regarding any missing ratings. The mean value of the ratings was displayed below the applicant’s photograph (Fig 1) to allow for comparison across applicants. Participants in the unsystematic and somewhat systematic condition were not provided with the criteria, sliders and calculation of mean ratings. The module where they read the CVs consisted of the leftmost part of Fig 2 only.

### Job analysis

An important preparatory part of the job analysis was to make importance-ratings of the job content according to a predetermined set of questions. The questions were inspired by the work skills and knowledge instruments on the US Department of Labor’s Occupational Information Network, O*NET (www.onetonline.org) but were somewhat modified and shortened. Of the set of 32 questions (S1 File), half were highly relevant for the job and half were not, i.e. concerned areas that were less important for job performance.

Participants were first presented with the job description and asked to read it. In systematic conditions, they then received the 32 questions, one at the time, and responded on a five-point Likert scale ranging from "not at all important" to "very important, while having access to the job description. When they were finished, their scores on the 6 criteria were displayed in a separate window. To minimize confusion only scores on the relevant job factors were displayed. For participants in the highly systematic condition, these factors were also displayed when reading the CVs. The job analysis step was included to make the recruitment process more transparent.

To ensure that the participants did not skip the job analysis it was set to a minimum time of 9 minutes. If they spent less time responding to the job analysis questions they were asked to read through the job description again and memorize central aspects of the job.

### Job and applicants

The job opening concerned a position as first-line supervisor (O*NET: First-Line Supervisors of Retail Sales Workers) for a furnishing store company. To maximize validity, we used those six skills and characteristics that were listed as most valuable for the job by the O*Net database, and wrote an ad and a job description (S1 File).

The applicants’ CVs were constructed so that they would differ in job-related characteristics, particularly with regard to the extent that their competence fitted the job description. The main measure for evaluating the hypotheses was quality of selection, i.e. the mean competence of the selected candidates. There were also process measures included, we measured time (reading CVs), and reliance on ratings in selection decisions.

## Study 1

Arguably, systematicity may be particularly beneficial when the recruiter is unaware of the full contents of the job and when the group of applicants appears homogeneous with regard to qualifications. In the present research, the applicants will have approximately the same general level of qualifications, and in this sense be akin to those real-world applicants who have passed the first round of screening. This should make it relatively difficult to differentiate them. However, they will differ with regard to how well their specific qualifications fit to the job that they apply for. Those in the high competence level group will be stronger on highly job-relevant criteria and weaker on the less relevant criteria, whereas those in the lower competence level group will be weaker on highly job-relevant criteria and stronger on less relevant. This way, all applicants will appear to be competent people, although some of them will be less competent in relation to the specific job.

The experimental design will include three different conditions; the unsystematic (level 0), the somewhat systematic (level 1) and the highly systematic (level 3). It is predicted that a more systematic approach results in selection of applicants with higher average competence.

### Method

#### Participants

The number of participants was 256; 64 in the unsystematic condition, 64 in the somewhat systematic condition and 128 in the highly systematic condition (the fact that that there were twice as many participants in the highly systematic condition was due to a malfunctioning of the software, half of these participants were supposed to belong to the systematic condition, but the software selected them to the highly systematic condition). The design of the experiment was such that the sample size had to be a multiple of 32. Since we aimed for medium-sized effects, we estimated that 64 participants would be required. Most participants were students, recruited by an experimenter on the campus of Lund University, and their mean age was 24.1 (*SD* = 3.8). They volunteered to participate in the study and received a movie ticket as compensation.

#### Materials and procedure

The CVs were created in the following steps: First, a large number of sentences were created. Each sentence provided information about the applicant’s level on one of 12 different job competencies, half of which were highly relevant for the job and half of which were less relevant. The highly relevant were: Establishing and maintaining interpersonal relationships, Speaking, Coaching and developing others, Stress tolerance, Communicating with superiors, colleagues or subordinates, and Self-control. The less relevant were: Performing for or working directly for the public, Training and teaching others, Interpret the meaning of information for others, Analyze data or information, Active learning, and Performing administrative activities.

In the second step, a sample of members of the same population as the participants rated these sentences in relation to how competent they believed the person described was. The outcome of the two first steps was a large number of descriptions that varied in level of general competence but also to what extent they were relevant for the job (specific competence).

In the third step, the sentence ratings were combined with their O*NET grading to create combinations of sentences that varied both regarding their level of relevance and their level of competence. Three kinds of combinations of sentences were created, one with sentences describing high competence on the job-relevant kind and lower competence on the less job-relevant kind, one with a medium level of both kinds of competence, and one with low competence on the job-relevant kind and high on the less job-relevant kind. This way, we ended up with combinations of sentences that had the same general level of competence, but with three clearly separated levels of relevance. All applicants had both relevant and less relevant competencies (three of each) but varied in the extent to which their relative strengths were on the relevant or less relevant side.

Applicants were presented with their photo and name. Their relative location on the screen was completely random and new for each participant. All 12 applicants were about the same age, between 25–40 years. There were six women and six men, six attractive and six unattractive. The competence level was varied across all applicants. The different CVs were balanced in a way such that all applicants (photos) were presented together with all CVs. The ideal selection was the four candidates that fitted the job best.

### Results and discussion

Descriptive data are provided in Table 1, and give a picture of the participants’ general performance in the selection task. There was a clear difference in competence between selected and not selected applicants, across conditions, *t*(256) = 14.221, *p* < .01. For the unsystematic condition, this difference was just barely significant, *t*(64) = 2.880, *p* < .01, *d* = 0.12, while it was larger for the somewhat systematic condition, *t*(64) = 6.347, *p* < .01, *d* = 0.78 and comparatively large for the highly systematic condition *t*(128) = 15.921, *p* < .01, *d* = 1.41. According to the hypothesis, participants in the highly systematic condition were expected to select applicants with a higher mean level of job-relevant competence than participants in the two less systematic conditions. To test this a one-way ANOVA was performed, and the results (see Table 1) indicated a clear difference between the conditions, *F*(2, 253) = 16.821, *p* < .01. Post-hoc testing revealed that all three conditions were separated. The selection quality was better in the somewhat systematic condition than in the unsystematic condition, *t*(126) = 2.031, *p* <. 05. It was also better in the highly systematic than in the unsystematic condition, *t*(190) = 5.61, *p* < .001. Finally, selection quality was higher in the highly systematic condition than in the somewhat systematic condition, *t*(190) = 3.26, *p* = .001. Accordingly, the main hypothesis was supported by the result, showing that increased systematicity causes a higher selection quality.

**Table 1:** Descriptive statistics for Study 1.

	Unsystematic		Somewhat systematic		Highly systematic	
	Mean	*SD*	Mean	*SD*	Mean	*SD*
Selection quality	2.132	.358	2.242	.305	2.402	0.278
CV reading time (s)	665	343	680	282	1116	395

*Note*. Quality of applicants varies from 1 to 3, with 2 suggesting the selection to be random.

Regarding the process-related variables, participants in the highly systematic condition spent clearly more time reading the CVs than participants in the somewhat systematic condition. Interestingly, reading time correlated positively with selection quality, *r*(254) = .295, *p* < .001. We analyzed whether the time spent on reading CVs mediates the difference between the conditions and found that it partially did, it was a significant covariate, *F*(1, 252) = 6.626, *p* < .05. Although the remaining difference was smaller, *F*(2, 252) = 6.649, *p* < .01, it was still clearly significant. The difference between the highly systematic condition and the unsystematic condition was clearly significant (*p* < .001) even after controlling for reading time, whereas the difference between the highly systematic and the somewhat systematic condition was marginally significant (*p* = .073). This suggest that the time that participants in the highly systematic condition put into CV-reading partially explains their higher performance. In addition, the correlation between CV reading time and selection quality in the highly systematic condition was significant, *r*(126) = .188, *p* < .05, suggesting that the time each individual spends on CV-reading affects selection quality.

We also examined whether participants in the highly systematic condition actually used their ratings from the CV-reading when making the final selection of the candidates. It was found that the more they relied on their ratings of the candidates when making the selection, the higher the selection quality, *r*(120) = .207, *p* < .05 (some of the ratings were missing because of malfunctioning of the software). This indicates the importance of adhering the task procedure.

In Study 1 the mean competence of selected applicants was clearly higher when the selection process was based on systematic compared to unsystematic methods. However, the study also indicated that those in who engage in systematic selection need quite a lot of support to succeed. This suggests that that certain conditions must apply if a systematic approach is to function well, and outdo an unsystematic approach.

## Study 2

To further probe what the level of systematicity needed in order to be able to select the best applicants, Study 2 tests whether selection performance is dependent on the rating tool introduced in the previous studies, or if performance is intact for participants who do not have access to it. In other words, we compare the systematic (level 2) with the highly systematic (level 3) condition. Our general prediction remains the same, increased systematicity is expected to enable better selection decisions. Furthermore, Study 2 increases the focus on process variables, namely CV reading time, and how motivation relates to selection quality.

The finding from Study 1 that the time spent on reading CVs is positively related to selection quality suggests that the more seriously one takes the assignment the better the results. The variability in time spent on reading CVs also suggests that our experimental situation allowed some participants to exert less effort than they would have exerted if more were at stake. To follow up on this, in Study 2 the highly systematic condition had an instruction added intended to raise the perceived cost of being careless during CV-reading. Participants are told that if they do not perform the task successfully they might have to redo it, afterwards. We expect this motivation manipulation to increase time spent on the job analysis, increase selection quality, and to decrease the relation between time spent on CV-reading and applicant quality. In other words, to the extent that increased motivation pushes recruiters to perform their task carefully, overall performance should increase and the room for individual differences diminish.

### Method

#### Participants

There were 144 participants, mostly students recruited by an experimenter on the campus of Lund University, with a mean age of 23.2 years (*SD* = 3.35). They were assigned to three conditions with 48 persons each (since we aimed for medium size effects, and the sample size had to be a multiple of 12); somewhat systematic, systematic, and highly systematic with motivation manipulation. All participated on a voluntary basis and were compensated with a movie ticket.

#### Materials and procedure

The computer application from Study 1 was used again. The motivation manipulation was introduced immediately before the participants started reading CVs and rating applicants. It stated that:

"Your next task takes at least 30 minutes. It is important not to be careless when working on it. For the results of your effort to be useful, you need to reach a certain level of performance. If you are careless and do not reach a satisfactory level, you will unfortunately have to do the complete task again.”

### Results and discussion

Table 2 provides descriptive data on participants’ general performance in the selection task. In all conditions, participants selected candidates who were clearly better than average. The selected applicants’ mean competence was lower for the somewhat systematic condition, *t*(47) = 4.208, *p* < .001, *d* = 0.60, than for the systematic condition, *t*(47) = 8.291, *p* < .001, *d* = 1.19, and highest for the highly systematic condition *t*(47) = 18.728, *p* < .001, *d* = 2.70. The values of the somewhat systematic condition are well in line with the results of Study 1 whereas the values of the systematic condition are slightly below the highly systematic condition of that study.

**Table 2:** Descriptive statistics for Study 2.

	Somewhat systematic		Systematic		Highy systematic + motivation	
	Mean	*SD*	Mean	*SD*	Mean	*SD*
Selection quality	2.177	0.292	2.292	0.244	2.526	0.194
CV reading time (s)	617	224	650	257	1476	568

*Note*. Quality of applicants varies from 1 to 3, with 2 suggesting the selection to be random.

The main hypothesis was that participants in the systematic condition would make better selections than participants in the somewhat systematic condition. This result was just significant, *t*(94) = 2.089, *p* < .05, which supports the hypothesis that job analysis increases selection quality. There was also a significant difference between the mean competence of the selected applicants in the highly systematic condition in Study 1 (*M* = 2.40), and the systematic condition in Study 2 (*M* = 2.29), one-sample *t*(174) = 2.325, *p* < .05. These results indicate that both job analysis and rating devices promote selection quality.

The only difference between the somewhat systematic and systematic condition in this study was that in the latter condition participants conducted a job analysis before selecting the applicants. It seems reasonable to expect that the time spent on reading CVs should differ between the systematic and somewhat systematic condition. However, there was no such effect, *t*(94) = 0.656, *p* > .05, suggesting that participants in the two conditions spent a similar amount of time on reading the CVs. The CV reading time correlated positively with selection quality, *r* = .283 *p* < .01. The correlation was low in the somewhat systematic condition (*r* = —.064, *p* > .05), but relatively higher in the systematic condition (*r* = .240, *p* < .05). This hints to a hypothesis worth testing in the future; only participants who work systematically are helped by spending more time reading the CVs.

The highly systematic condition showed the highest selection quality of all. A one-sample *t*-test showed that the selection quality was clearly higher than in the highly systematic condition in Study 1, which had no motivation instruction, *t*(47) = 4.416, *p* < .001, *d* = 0.63 (using the sample *SD* of this condition). We had hypothesized that the correlation between quality and reading time would decrease as an effect of the motivation manipulation, since there is less room for individual differences. Indeed, the correlation was found to be almost zero, *r*(46) = -.051, *p* > 05. Furthermore, as in Study 1, participants who followed their CV-reading ratings performed somewhat better than those who tended depart from their ratings, *r*(46) = .169, but perhaps because the study had lower power it was not significant (*p* > .05) although the correlation strength was almost identical to that of Study 1.

## General discussion

The present research was an attempt to investigate selection-related decision making in the laboratory with an experimental paradigm. The experimental setup enabled experimental study of personnel selection while tracking process related variables. Crucially, level of systematicity was varied in a stepwise fashion. We applied different tools in order to make the process more systematic and investigated if this made a difference in the selection of job-applicants. Increasing systematicity, especially when a job analysis was supplemented by a rating tool, led to better selection decisions, particularly when the motivation to put some effort into the task was increased too.

### Contributions to selection research

#### Selection research in general

In the present research, all participants were given the same information about the applicants and almost all were given the same information about the job, but they processed it differently. Increasing systematicity by providing a job analysis tool and a rating tool enabled somewhat better decisions than when working less systematically. This goes beyond the results of previous related research, which has had more proximal outcome variables. Furthermore, the present research differs in that the focus was not on a systematic vs. unsystematic version of a specific selection tool. Rather, it regarded the approach of how to treat information in a selection context. The results showed convincingly that more thoroughly and methodically (i.e. systematically) the task was approached, the better the result.

Our results on the effects of systematicity on selection decisions are in line with what many researches in the area (e.g. Highhouse [1]) recommend (e.g. *Principles for the validation and use of personnel selection procedures* from Society for Industrial and Organizational Psychology and ISO 10667). We take our findings as lending further credibility to these recommendations. Our results constitute direct evidence of the merits of decision tools in selection. Failure to use them is likely to lower decision quality. As suggested by Highhouse [1] recruiters may have personal motives to compromise with the use of selection decision tools. According to our results, such compromises may come with a cost.

Nothing in our results suggested that hybrid forms of information processing (compromising with systematicity) are to be preferred in the current selection context. Granting that our study was optimized to test the merits of different levels of systematicity rather than intuitive processes, we note that increased selection quality was related to increased (rather than decreased) systematicity. The present results are congruent with a linear model of the effect of systematicity on selection quality, but do not exclude that hybrid effects [8] may appear in other selection contexts.

#### Job-analysis

Previous research has shown that training leads to increased accuracy and reliability in competency modeling [13], job evaluations [19] and performance appraisal [20]. It has also shown that having the ratings concern specific tasks rather than the job as a whole generally improves job analysis ratings, particularly after training [12]. These findings are in line with the general argument in the current study, namely that systematic selection is generally more valid and reliable than less systematic selection. We hasten to add that just doing a job analysis does not seem to have any large influence, it is when the criteria from the job analysis are used in the decision process in a systematic fashion that quality improves.

In a recent related study, Aguinis, Mazurkiewicz and Heggestad [14] probed the effects of job analysis on the personnel selection process. They developed a job analysis tool that resembles ours in that the purpose was to make the recruiter focus on job related aspects, and used it in a field experiment. The results showed that when recruiters use a job analysis instrument, the correlation between their ratings of which traits are important for the job and ratings of which traits they possess themselves decreases. In other words, it appears that the job analysis instrument helps recruiters to avoid overemphasis on traits that they happen to possess themselves, and instead focus on what the work-task affords. Specifically, the risk of hiring candidates that are similar to oneself should decrease. In any case the Aguinis, Mazurkiewicz and Heggestad [14] research is impressive in that it not only identified a bias but also showed that job analysis mitigates it. However, in contrast to our study, there was no actual selection of candidates, only ratings of trait importance. Both studies point to the importance of job analysis in selection. The Aguinis, Mazurkiewicz and Heggestad study identified a way to reduce a bias that may result in exclusion of candidates that in fact ought to be selected. Our study rather suggests that job analysis is a necessary preparatory step when performing a high quality selection, but its main role is to focus the selection process on valid worker attributes. To use these attributes when comparing the applicants in a systematic fashion seem at least as important.

#### Inferring worker attributes

The present research compared the quality of worker attribute inferences for more vs. less systematic selection. We expected that being provided with a rating tool would allow better discrimination of applicants on their actual level of qualification. In other words, having access to a rating tool should enable selection of candidates that are more suited for the job than when not having access to such a tool. This was also found to be the case. Since our results regarding the rating tool concern selection decisions they add to previous research which has showed that differentiating the job analysis items such that they concern the specific tasks that the job involves rather than the job as a whole generally improves the validity of job analysis ratings [12]. Our rating tool appears to have facilitated application of relevant criteria when making judgments about applicants’ attributes. The results show that worker attribute inferences were improved, and resulted in better selection decisions. Interestingly, those who compromised with their own systematically derived worker attribute inferences (ratings of applicant’s CV-summaries) made worse selection decisions than those who trusted their preceding inferences and went along with them. This is further evidence of the benefits of sticking to systematicity, rather than compromising with it.

#### Task motivation

The benefits of systematicity were particularly clear when those who performed the selection task were motivated to do it well. In Study 1 it was found that participants who spent more time on the selection task also made better selection decisions. If time spent on the task had been inversely related to success, individual differences in cognitive capacity would have been a viable explanation. However, since spending more time was positively related to success, motivation was assumed to be the underlying factor, and it was tested in a more direct fashion in Study 2. Informing the participants that they may have to redo the complete task if the selection was unsatisfactory in led to a better selection. This result is in line with recent research on job-analysis judgments [21], where the difference between holistic and decomposed job-analysis judgments increased when raters were careless (as measured by endorsement of bogus items). Since our dependent measure regarded selection quality rather than job-analysis judgments, the present results contribute by linking motivation and systematicity to selection outcome. The job-analysis literature shows that respondents differ in how carefully they read items, whether they respond appropriately to what is asked for, and whether they make distinctions between items that should be distinguished [22]. The present results suggest that these individual differences will reveal themselves in selection decisions too. Since the motivation manipulation was only introduced in the systematic condition we cannot tell whether it is also beneficial in selection situations with lover levels of systematicity, and confine ourselves to concluding that systematic selection is dependent on the recruiters being motivated to perform the tasks in the manner that they were designed to.

### Practical implications

In the present study we attempted to design a novel experimental task which brings the researcher closer to how an actual selection process is performed, including process related variables such as time spent on CV-reading. This was rendered possible by a computer application, a very flexible research tool. Computer applications may be used when probing other important research questions, e.g. simulating scenarios with an increased risk for discrimination. Furthermore, their scope may be increased by adding other parts, such as the interview. They may also be used as educational tools for training recruiters. It is conceivable that first-hand experience of the benefits of increased systematicity may motivate further use of related methods.

### Limitations

Our results primarily concern the extent to which unexperienced recruiters are helped by having access to a job analysis tool and a rating tool. Such results are relevant, since many times selection tasks are performed by persons with little or no training. However, it is a limitation of the study that there were no experienced recruiters among the participants. Instead, in both experiments we used a convenience sample of university students, a group in which virtually none has experience from recruitment, as opposed to professionals, who are more experienced (although the level of experience may vary considerably amongst them). We believe that professional recruiters too are helped by decision tools, but that it is possible that they perform better than inexperienced recruiters when for some reason (e.g. lack of documentation, lack of time, etc.) it is necessary to compromise with systematicity. However, personnel selection is not a repetitive routine task. The possibility to acquire rules through implicit learning or automatize the process in some other way is not large. This is one reason why the role of expertise should not be exaggerated and studies actually indicate that it may be the experienced recruiters that benefit the most from systematicity [13, 21]. Even experts have a hard time making good selection decisions, especially when there is a variation in the jobs they recruit to.

Even if we have attempted to create a computer application that shares many important features with a real selection task, there are of course differences between a laboratory study and an actual selection process. One important difference is that real selection decisions should involve a stronger sense of accountability, since one may be asked to justify one’s judgments and decisions to the applicant, the employer or even a representative of the legal system. This is difficult to reproduce in the laboratory, where one generally is responsible towards the experimenter rather than the employer. We don’t see accountability concerns as threatening the internal validity of our findings, but suggest that future studies should consider accountability motivation in order to maximize their external validity.

Finally, the selection decisions were evaluated in relation to established criteria (from O*Net), not actual job performance. In other words, although the computer application can be used to investigate to what extent different tools help improve selection decisions, it is not to be seen as a selection tool itself. Its use is limited to evaluation of decision tools and as part of a package for training recruiters in systematic selection and recruitment procedures.

## Conclusions

What is the relevance of studying the effects of level of systematicity on decisions in a computer application related to personnel selection? The current research showed the effects of systematicity on selection related judgments and decisions, and the studied process variables contributed a better understanding of how the two decision tools provided can help recruiters make better selection decisions. The studies testify to the value of performing research in the intersection between the applied and the cognitive-experimental domain. Experimental studies generally offer more control and increased possibilities of drawing causal conclusions. Such data may contribute to fill an apparent gap in the personnel selection literature, where many studies have concerned systematicity but few have provided direct comparisons of the outcome-related benefits of different levels of systematicity. These results are in line with what Industrial/Organizational psychologists have advocated for years, but add to them and may prove useful in the work with implementing higher levels of systematicity in actual personnel selection.

## Supporting information

S1 File The job analysis questions, job advertisement, job description, and examples of CVs. (DOCX)
