# Supplementary material for: Increasing systematicity leads to better selection decisions: Evidence from a computer paradigm for evaluating selection tools
Source: PLoS One. 2017 May 22;12(5):e0178276. doi: 10.1371/journal.pone.0178276 (PMC5440047; doi:10.1371/journal.pone.0178276)
Supplement: S1 File — (DOCX) [file pone.0178276.s001.docx]

**S1**

**Stimulus materials (all translated from Swedish)**

*The job analysis questions:*

How important is it to understand what others are saying to succeed at the job?

How important is it to have a rich flow of ideas for the job performance?

How important is it to be able to express oneself orally for the job performance?

How important is it to be good at deductive reasoning (drawing conclusions from general rules) for the job performance?

How important is it to be sensitive to problems that may emerge at the workplace for the job performance?

How important is it to be skilled in inductive reasoning (drawing conclusions from the specific to the general) for the job performance?

How important is it to have talent for oral communication with colleagues and superiors?

How important is it to have administrative and leadership skills for the job performance?

How important is it to be good at collaborating with others for the job performance?

How important is it to be innovative for the job performance?

How important is it to have self-control to accomplish the work tasks well?

How important is it to be proficient in analytic thinking to carry out the job?

How important is it to be able to carry out discussions well with individuals and groups?

How important is it to take responsibility for the health and security of other employees to be successful in the job?

How important is having contact with other people at work (directly, over the telephone, or otherwise)?

How significant is it to be able to acquire much information for the job performance?

How important are interactions where you have to work with (or contribute to) a work group or team for the job performance?

How significant is it to be proficient in processing and treating information for the job performance?

How important is it to be able to take criticism and not lose ones temper if somebody has opinions on how the work is carried out?

How significant is it to be capable of analyzing data or information for the job performance?

How significant is it to be able to communicate with superiors, colleagues, and subordinates for the job performance?

How significant is it to be good at thinking creatively for the job performance?

How significant is it to be able to establish and maintain interpersonal relationships for the job performance?

How significant is it to be focused on updating and using relevant knowledge for the job performance?

How significant is it to be good at guiding, directing and motivating subordinates for the job performance?

How important is it to have the capacity cope with a working environment which sometimes is demanding and where the standards are high?

How significant is it to have eye for recruitment and to select good personnel to the organization for the job performance?

How significant is it to have the ability to understand and communicate the meaning of information for the job performance?

How significant is it to be proficient in managing and overseeing other personnel for the job performance?

How significant is it to be able to endure periods of high workload?

How significant is it to be prepared to provide consultation and advice to others for the job performance?

How significant is it to be skilled in administrative tasks for the job performance?

*The job advertisement:*

**First line supervisor (sales manager)**

We seek a first line supervisor for our chain of stores, which in Malmö consist of 5 stores and approximately 40 salespersons. As head of the sales organization you will be responsible for the recruitment and introduction of salespersons, and you will also work together with other sales managers in the Southern region.

At work you will be the hub for all salespersons in Malmö, and your task is to make sure that all stores are staffed and that the salespersons get the support they need to do their job. You will work in close cooperation with the central marketing office and also with the company’s administrative staff, such as the salary office and the purchase department. We expect you to have some previous sales experience and it is also good if you have held a supervisory position before. Previous business education is a plus, but no requirement, since you will receive some in-service training.

*The job description:*

**Job as a First line supervisor (sales manager)**

As sales manager you are the hub of the sales organization. You work towards customers, stores and their salespersons, other divisions within the region as well as the company’s regional management.

The job involves planning for and carrying out recruitment of salespersons, providing support and supervision for employees, and cooperating with the marketing office so that campaigns etc. are synchronized regarding supply of goods and personnel. Moreover, the sales manager is expected to communicate with the accounts department and make sure that they have continuous access to essential information, and participate actively in meetings with other sales managers, under the leadership of the regional sales division head. When there are complaints directed at salespersons or stores it is the sales manager’s responsibility to do what can be done to discuss the problems that have arisen and find solutions to them. A large part of these tasks imply oral communication. It is thus important to be able to establish and maintain good connections to customers, employees and superiors. Periodically, the job involves a high workload and stress.

Further, it is the sales manager’s task to supervise the salespersons, support them, provide sales-advice or tips on how to handle particular types of customers, and provide support during stock taking. In more complicated sales situations, where the salespersons need support, the sales manager should have direct contact with customers, respond to their questions and meet those who have complaints regarding services or products. In sum, it is the sales manager’s responsibility to coach the individual salesperson and create opportunities for personal growth and development.

**Abilities**

The sales manager should have a number of qualities to perform the job optimally. The job involves informing different groups on a regular basis. It is therefore important to be able to convey messages efficiently so as to make oneself understood. For the sales manager to be able to establish and keep up relationships to persons at different levels, not only communicating skills are required, but also a strong interest in networking. Since much communication is oral it is important to be able to make oneself understood and efficiently inform others.

One of the most important abilities is to see which needs for development that the individual salesperson has. It is also imperative to be skilled in supervising, and supporting the salesperson to thrive in the sales role.

In the job challenging situations will come up, which demands very good self-control and ability to keep emotions in check, e.g. in handling dissatisfied and difficult customers. Moreover, the sales manager has a central position in the organization and therefor has to be good at handling claims and criticism which may come from many directions. It is therefore important handle stress well, particularly during the periods (e.g. with Christmas approaching) when the organization is under a heavy workload.

*Examples of CV-summaries:*

*A CV-summary at the low level of job-relevant competence*

X has experience of several jobs in the service industry and has received very strong recommendations. The references emphasize his/her ability to take care of guests and customers in different contexts. According to the interview, X sometimes sees difficulties in forming collaborations with everybody who is involved in the organization; from experience one works better with some people than with others, which must be taken into account when putting together a work group. On a direct question X feels that in-service training is important for the organization to become successful. He/She has positive experience from several courses. One of X’s references mentions his/her ability to perform work tasks such that the process becomes clear and tangible to those involved. He/She also gives the impression of being willing to take on tasks related to communication between different parts of the organization.

During the interview, it becomes clear that X has never been a mentor, but that he/she thinks that coaching is important in working life. There is strong consensus among various sources that X is very good at conveying a context, and to in the presence of others explain how complex relationships really work, a task where he/she evidently excels.

*A CV-summary at the medium level of job-relevant competence*

X has a very strong ability to express himself/herself orally and really knows how to convey a message so that he/she is understood regardless of audience. At the previous workplace it was particularly important to develop good working relationships with others, and according to referees X had certain success in this. Moreover, X has some experience of service work and has been commended for his/her ability to take care of customers. X has a relatively good ability to cull out the most important aspects of the information given and is also, and finds it stimulating, to convey this to colleagues.

X is rather capable of handling negative emotions in difficult situations and is described by a referee as a person with good self-control. Finally X emphasizes how important it is to learn whether the colleagues can become better at their tasks though more training etc., and would be pleased to familiarize himself/herself with the relevant training opportunities in this area and to enhance his/her own skills.

*A CV-summary at the high level of job-relevant competence*

X has on several occasions been asked to serve as instructor when the company has implemented in-service training. There are clear indications that this is an area which he/she both enjoys and is good at. X has also for many years acted as mentor in different contexts, and several references highlight his/her good ability to coach and develop others. The references emphasize that X is genuinely interested in good cooperation with colleagues and superiors. He/She is very adept at creating this kind of permanent and constructive relations. Furthermore X states a genuine interest in customer-oriented work, but has only short practical experience of this field.

At former jobs he/she has been confronted with information that has been very difficult to interpret and created difficulties for the work group. This is a situation which according to X arises often and which he/she hopes to be able to help with in the future. X has sometimes been asked to give oral presentations, and according to references he/she accomplished this well.
